# PCV7- and PCV10-Vaccinated Otitis-Prone Children in New Zealand Have Similar Pneumococcal and *Haemophilus influenzae* Densities in Their Nasopharynx and Middle Ear

**DOI:** 10.3390/vaccines7010014

**Published:** 2019-01-31

**Authors:** Camilla de Gier, Caitlyn M. Granland, Janessa L. Pickering, Tony Walls, Mejbah Bhuiyan, Nikki Mills, Peter C. Richmond, Emma J. Best, Ruth B. Thornton, Lea-Ann S. Kirkham

**Affiliations:** 1 School of Medicine, University of Western Australia, Perth 6009, Australia; camilla.degier@uwa.edu.au (C.d.G.); mejbah.bhuiyan@uwa.edu.au (M.B.); peter.richmond@uwa.edu.au (P.C.R.); ruth.thornton@uwa.edu.au (R.B.T.); 2 Wesfarmers Centre of Vaccines and Infectious Disease, Telethon Kids Institute, Perth 6009, Australia; caitlyn.granland@telethonkids.org.au (C.M.G.); janessa.pickering@telethonkids.org.au (J.L.P.); 3 Department of Paediatrics, University of Otago, Christchurch 8011, New Zealand; tony.walls@otago.ac.nz; 4 Starship Hospital, Auckland 1023, New Zealand; Nikki@adhb.govt.nz (N.M.); e.best@auckland.ac.nz (E.J.B.); 5 School of Medicine, University of Auckland, Auckland 1023, New Zealand; 6 Department of General Paediatrics, Perth Children’s Hospital, Perth 6009, Australia; 7 Centre for Child Health Research, University of Western Australia, Perth 6009, Australia

**Keywords:** carriage density, nasopharynx, New Zealand, otitis media, pneumococcal conjugate vaccine, NTHi, qPCR

## Abstract

Otitis media (OM) is a major reason for antibiotic consumption and surgery in children. Nasopharyngeal carriage of otopathogens, *Streptococcus pneumoniae* and nontypeable *Haemophilus influenzae* (NTHi), is a prerequisite for development of OM, and increased nasopharyngeal otopathogen density correlates with disease onset. Vaccines can reduce or eliminate otopathogen carriage, as demonstrated for pneumococcal serotypes included in pneumococcal conjugate vaccines (PCV). The 10-valent PCV (PCV10) includes an NTHi carrier protein, and in 2011 superseded 7-valent PCV on the New Zealand Immunisation Program. Data are conflicting on whether PCV10 provides protection against NTHi carriage or disease. Assessing this in otitis-prone cohorts is important for OM prevention. We compared otopathogen density in the nasopharynx and middle ear of New Zealand PCV7-vaccinated and PCV10-vaccinated otitis-prone and non-otitis-prone children to determine PCV10 impact on NTHi and *S. pneumoniae* carriage. We applied qPCR to specimens collected from 217 PCV7-vaccinated children (147 otitis-prone and 70 non-otitis-prone) and 240 PCV10-vaccinated children (178 otitis-prone and 62 non-otitis-prone). After correcting for age and day-care attendance, no difference was observed between NTHi density in the nasopharynx of PCV7-vaccinated versus PCV10-vaccinated otitis-prone (*p* = 0.563) or non-otitis-prone (*p* = 0.513) children. In contrast, pneumococcal nasopharyngeal density was higher in PCV10-vaccinated otitis-prone children than PCV7-vaccinated otitis-prone children (*p* = 0.003). There was no difference in otopathogen density in middle ear effusion from PCV7-vaccinated versus PCV10-vaccinated otitis-prone children (NTHi *p* = 0.918; *S. pneumoniae p* = 0.415). When pneumococcal carriage was assessed by vaccine serotypes (VT) and non-vaccine serotypes (NVT), there was no difference in VT density (*p* = 0.546) or NVT density (*p* = 0.315) between all PCV7-vaccinated versus all PCV10-vaccinated children. In summary, PCV10 did not reduce NTHi density in the nasopharynx or middle ear, and was associated with increased pneumococcal nasopharyngeal density in otitis-prone children in New Zealand. Development of therapies that prevent or reduce otopathogen colonisation density in the nasopharynx are warranted to reduce the burden of OM.

## 1. Introduction

Otitis media (OM) is the most common paediatric infection for which medical care is sought, and the most common indication for prescription of antibiotics in children [1,2]. OM is also one of the most common reasons for children to undergo surgery for ventilation tube insertion (VTI), with recurrent acute OM (rAOM) or persistent otitis media with effusion (OME) being the usual indications. Hence, OM places a significant burden on healthcare systems and on affected families [3,4,5]. Most episodes of severe OM are from bacterial causes, with the most common species involved being *Streptococcus pneumoniae* (pneumococcus) and nontypeable *Haemophilus influenzae* (NTHi). Nasopharyngeal colonization with otopathogens is considered to be a prerequisite to development of disease [6,7,8,9,10,11]. Vaccines that target these pathogens have the potential to reduce nasopharyngeal carriage and transmission, leading to a reduction in the prevalence of OM and in turn to a reduction in antibiotic prescriptions and children undergoing VTI surgery. *S. pneumoniae* is the only otopathogen that is directly targeted by currently licensed vaccines. Introduction of pneumococcal conjugate vaccines (PCV) has reduced the incidence of OM from the serotypes included in the vaccine [12,13,14], but has had a limited impact on the overall prevalence of OM due to replacement disease from non-vaccine serotypes and other otopathogens [15,16].

The 7-valent PCV (PCV7) was introduced into the New Zealand Immunisation Program in 2008, and national surveillance reports showed a significant decrease in the rate of invasive pneumococcal disease in children <5 years of age in the following years [17]. In 2011, PCV7 was replaced by the 10-valent PCV (PCV10), which covers 3 additional pneumococcal serotypes and has Protein D from *H. influenzae* as the conjugate protein for 8 of the 10 serotypes [18,19]. From an OM prevention point of view, it is of particular interest to assess whether the addition of Protein D influences the nasopharyngeal carriage and development of OM from NTHi. A recent Phase IV clinical trial in Australian Aboriginal children, who have a high risk of chronic OM, showed that PCV10 vaccination reduced the prevalence of NTHi detection in middle ear discharge when compared with PCV7, although no difference in nasopharyngeal NTHi carriage rates was observed [20]. Likewise, studies from the Netherlands and Finland, including non-otitis-prone children with a lower risk of developing NTHi OM, showed that PCV10 vaccination had no impact on NTHi carriage rates [21,22]. In these studies, carriage was assessed by culture of nasopharyngeal swabs, with presence or absence of an otopathogen reported rather than the density of otopathogens. It has been suggested that quantitative analysis, rather than culture, should be used to determine the concordance between carriage and disease [23]. Quantitative (q) PCR was previously applied to nasopharyngeal swabs in an Australian study, and revealed that children with chronic OM had a significantly higher median load of otopathogens in their nasopharynx compared with non-otitis-prone children. They also observed that Australian Aboriginal children had significantly higher nasopharyngeal densities of otopathogens than their non-Aboriginal counterparts [24]. Recently, an animal model of AOM has demonstrated that vaccination with Protein D, the NTHi carrier protein in PCV10, reduced NTHi density in the middle ear but had no impact on density of NTHi in the nares [25]. Whether this occurs in PCV10-vaccinated children has not been investigated before, but may explain the findings of the Leach et al. study [20], where a reduction in NTHi-associated OM was observed without reduction in NTHi carriage rates following PCV10 immunisation.

To determine the aetiology of OM in New Zealand in the PCV7 era, nasopharyngeal swabs and middle ear effusions were collected from 325 PCV7-vaccinated otitis-prone children and 137 PCV7-vaccinated non-otitis-prone children, with NTHi identified as the predominant otopathogen detected by culture and PCR (non-quantitative) in the collected specimens [26]. In 2014, three years after the introduction of PCV10, a second cohort of 319 PCV10-vaccinated otitis-prone children and 154 PCV10-vaccinated non-otitis-prone children were recruited [27] to compare OM otopathogens with the PCV7-vaccinated children. NTHi remained the predominant otopathogen identified in nasopharyngeal swabs and middle ear effusions from the PCV10-vaccinated children, with both NTHi and *S. pneumoniae* carriage rates remaining unchanged in the two vaccine cohorts [27]. To further assess PCV10 impact and aid development of future OM therapies, we have applied qPCR to specimens collected in these cohorts to (1) determine whether PCV10 vaccination reduces NTHi density in the nasopharynx and middle ear of otitis-prone children, and (2) assess the impact of PCV10- versus PCV7-vaccination on pneumococcal carriage density in otitis-prone and non-otitis-prone children.

## 2. Methods

### 2.1. Nasopharyngeal Swabs and Middle Ear Effusion Samples

A total of 457 nasopharyngeal swabs and 411 middle ear effusion samples were included in this study. Samples were collected as previously described [26] from children less than 36 months of age and either undergoing VTI surgery for rAOM/OME (otitis-prone cases) or surgery for non-infectious reasons (non-otitis-prone comparison group; controls). Recruitment for the PCV7 vaccine group occurred between May to November 2011, and for the PCV10 vaccine group between May to November 2014. All children were fully PCV-vaccinated according to the National Immunisation Program for New Zealand. Season- and age-matching was conducted for recruitment of controls. Additional exclusion criteria for this study included cases that had only middle ear effusion collected and no matching nasopharyngeal swab. Nasopharyngeal swabs and middle ear effusions were collected and stored as previously described [28].

### 2.2. DNA Extraction

Stored samples were thawed, vortexed thoroughly for 30 seconds, and material transferred to RNase free microcentrifuge tubes for enzymatic lysis. Tubes were centrifuged at 13,000× *g* for 7 min and supernatant was discarded. The pellets were resuspended in 180 μL enzymatic lysis buffer (20 mM Tris-HCL pH 8.0, 2 mM Na-EDTA pH 8.0, 1% Triton X-100 (Sigma-Aldrich, Castle Hill, NSW, Australia), 2 mg/mL RNase A (Thermo Fisher Scientific, Scoresby, VIC, Australia), 0.075 mg/mL Mutanolysin (Sigma-Aldrich), 20 mg/mL lysozyme (Sigma-Aldrich) plus 5 μL of internal DNA Extraction Control (IC) (Bioline, Alexandria, NSW, Australia). After incubation at 37 °C for 60 min, 20 μL of 20 mg/mL Proteinase K (Qiagen, Chadstone, VIC, Australia) and 200 μL Buffer AL (Qiagen) was added and samples were incubated at 56 °C for 30 min. After the final incubation step, samples were stored at −20 °C until the following day. DNA was extracted from the lysed samples using the Qiacube HT (Qiagen). A negative extraction control consisting of STGGB only was included in each extraction run. Genomic DNA was stored in aliquots at −80 °C for long term storage (and at −20 °C for no more than 6 weeks). Reference strain genomic (g) DNA was extracted from overnight plate cultures of NTHi 86-028NP [29] and *S. pneumoniae* NCTC 7466 using the QIAamp DNA mini kit (Qiagen) and following the manufacturer’s protocol. DNA concentrations of the reference strains were measured on the Qubit 3.0 Fluorimeter using the Qubit dsDNA BR Assay Kit (Thermo Fisher Scientific, Scoresby, VIC, Australia). The extracted reference strain gDNA was stored as single use aliquots at −80 °C, and DNA concentration remeasured on the Qubit before each use.

### 2.3. Quantitative PCR

Real-time qPCR was conducted using the CFX96 real-time PCR detection system (Bio-Rad, Gladesville, NSW, Australia). The qPCR primers, probes and reaction conditions are detailed in Table 1. The total reaction volume was 10 µL. The two genes used to detect NTHi were run together in a duplex qPCR assay, as previously described [30]. The reaction mix consisted of 5 µL of 2× SensiFAST Probe No-ROX (Bioline), 0.4 µL 25× internal DNA Extraction Control mix (Bioline), primer and probe as per concentrations listed in Table 1, molecular grade water (Sigma-Aldrich) and 1 µL of sample DNA. A standard curve was generated for each run using serial dilutions of gDNA (2000 pg to 0.02 pg) from each reference strain. Cycling conditions are described in Table 1. All samples were run in duplicate, and the density was calculated as an average of the two measurements. Analysis of the qPCR data was performed using Bio-rad CFX manager 3.1 (Bio-Rad). The internal control (IC) qPCR was run alongside the NTHi qPCR, and threshold cycle value (Ct) for each sample was compared with the Ct for the negative extraction control. Samples were considered accepted if within 1 Ct value on either side of the Ct for the negative extraction control. Samples that failed IC qPCR were re-run and excluded from further analysis if they failed IC qPCR twice. Specimens that were culture positive for *S. pneumoniae* were serotyped by either microarray [31] or Quellung [27], making it possible to assess serotype-specific carriage density based on the *S. pneumoniae* specific *lyt*A qPCR. We used the dominant serotype present in the specimen and matched this with the overall pneumococcal density.

### 2.4. Statistical Analysis

Samples with DNA densities below the limit of quantification (LOQ; 0.0125 pg/µL) were assigned half of this value (0.00625 pg/µL) rather than zero, to permit statistical analyses. Host and environmental risk factors between cases and controls were compared using Student’s t tests for continuous variables (age), and Pearson chi-square analyses (P value, asymptotic significant, 2-sided) for categorical variables (gender, day-care attendance, ethnicity, current antibiotic use). Binary logistic regression was used to determine the association between the presence of otopathogens in specimens and case/control status or vaccine recipient groups (PCV7 or PCV10), adjusting for the cofounding variables of age and day-care attendance to give the adjusted odds ratio (aOR). A linear regression model (on log-transformed densities and adjusting for confounders of age and day-care attendance) was used to compare density of otopathogens in positive specimens collected from PCV7- and PCV10-vaccinated otitis-prone and non-otitis-prone children. The significance level was considered as *p* < 0.05. Spearman’s rank correlation coefficient was used to determine the correlation between otopathogen densities in the nasopharynx and middle ear, where 0.5 > *r* > 0.3 was considered to be a weak correlation and 0.7 > *r* > 0.5 was considered to be a moderate correlation. The area under the receiver operating characteristic (AU-ROC) curve was determined to assess the potential value of a nasopharyngeal otopathogen density test for differentiating disease status (otitis-prone) from non-disease status (non-otitis-prone controls), where an AUC of 0.5 = no diagnostic ability, AUC 0.5–0.6 = poor diagnostic ability, AUC of 0.6–0.7 = sufficient diagnostic ability, an AUC 0.7–0.9 = good to excellent, and an AUC of 1.0 = perfect diagnostic ability. IBM SPSS Statistics version 22 was used for all statistical analysis. Graphs were plotted using GraphPad Prism 7 (GraphPad Software Inc., San Diego, CA, USA).

### 2.5. Ethics

The study was conducted in accordance with the Declaration of Helsinki, and the protocol was approved by the New Zealand Northern Regional Ethics Committee (NTX/11/04/029). Informed written consent was obtained from parents/guardians of study participants at the time of recruitment.

## 3. Results

### 3.1. Study Population

Of the 457 children included in this study, 217 were recruited in 2011 and were PCV7-vaccinated (147 otitis-prone cases and 70 non-otitis-prone controls), and 240 were recruited in 2014 and were PCV10-vaccinated (178 otitis-prone cases and 62 non-otitis-prone controls) (Table 2). All children included in this study were fully vaccinated with either PCV7 or PCV10 according to the National Immunisation Program. The controls were approximately 3 months younger than the cases in both vaccine groups. Day-care attendance was more common in the otitis-prone cases compared to the non-otitis-prone controls in both the PCV7-vaccinated (63% versus 40%; *p* = 0.002) and PCV10-vaccinated (81% versus 45%; *p* = 0.0001) groups. More of the otitis-prone children in the PCV10-vaccinated group attended day-care for >4 h/week in comparison with the otitis-prone children in the PCV7-vaccinated group (81% versus 63%, *p* = 0.001); whereas day-care attendance in the non-otitis-prone controls was similar between the vaccine groups, *p* = 0.270 (Table 3). While there were more children of Māori descent in the PCV10-vaccinated cohort than the PCV7-vaccinated cohort (Table 3), there was no difference in the proportion of otitis-prone versus non-otitis-prone Māori children in each vaccine group: PCV7-vaccinated otitis-prone Māori versus PCV7-vaccinated non-otitis-prone Māori children, 14% versus 7%, *p* = 0.130; and PCV10-vaccinated otitis prone Māori versus PCV10-vaccinated non-otitis-prone Māori children, 25% versus 23%, *p* = 0.671 (Table 2). Age and day-care attendance were adjusted for as confounders in all subsequent analysis.

### 3.2. No Difference in Otopathogen Presence in the Nasopharynx of PCV7- and PCV10-Vaccinated Otitis-Prone or Non-Otitis-Prone Children

There was no difference in the proportion of otitis-prone children colonised with NTHi between vaccine groups: 61.9% (91/147) for PCV7-vaccinated cases versus 68.5% (122/178) for PCV10-vaccinated cases, *p* = 0.283. However, the adjusted odds ratio (aOR) was 1.297, meaning that when the confounding factors of age and day-care were adjusted for, PCV10-vaccinated otitis-prone children were 29.7% more likely to carry NTHi than PCV7-vaccinated otitis-prone children. There was also no significant difference in presence of NTHi in the nasopharynx of non-otitis-prone controls from the different vaccine eras, with 40.0% (28/70) colonised with NTHi in the PCV7-vaccinated controls versus 56.5% (35/62) for PCV10-vaccinated controls, *p* = 0.207. However, the aOR was 1.652, meaning that PCV10-vaccinated controls were 65.2% more likely to be colonised with NTHi than the PCV7-vaccinated controls.

Similarly, there was no significant difference in the presence of *S. pneumoniae* in the nasopharynx of PCV7-vaccinated otitis-prone cases (55.8%, 82/147) versus PCV10-vaccinated otitis-prone cases 65.2% (116/178), *p* = 0.115. However the aOR was 1.452, meaning that PCV10-vaccinated otitis-prone children were 45.2% more likely to carry *S. pneumoniae* than the PCV7-vaccinated otitis-prone children. There was also no difference observed for the presence of *S. pneumoniae* in the nasopharynx between PCV7-vaccinated (40.0%, 28/70) and PCV10-vaccinated (48.4%, 30/62) non-otitis-prone controls (*p* = 0.619; aOR = 1.213), but PCV10-vaccinated controls were 21.3% more likely to be colonised with *S. pneumoniae* than the PCV7-vaccinated controls.

### 3.3. PCV10-Vaccinated Otitis-Prone and Non-Otitis-Prone Children Had Similar NTHi Nasopharyngeal Densities to PCV7-Vaccinated Otitis-Prone and Non-Otitis-Prone Children

**Figure 1:**
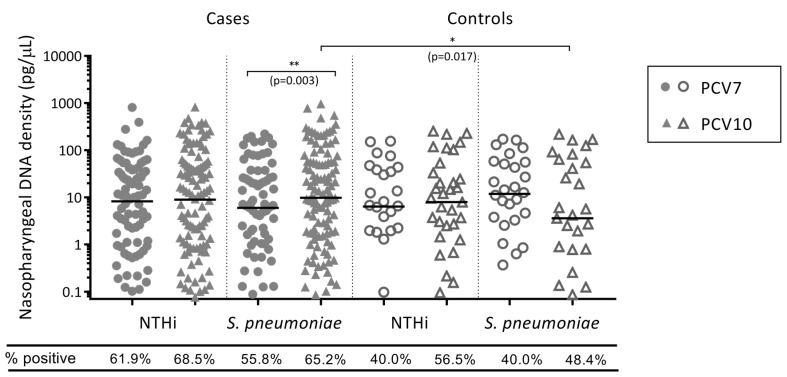
Otopathogen density in the nasopharynx of PCV7- and PCV10-vaccinated otitis-prone and non-otitis-prone children. Data are presented for children that were colonised with an otopathogen, with each point representing an individual child and the horizontal bars depicting the median otopathogen DNA concentration in pg/μL. Circles represent PCV7-vaccinated otitis-prone cases (closed circles) and non-otitis-prone control children (open circles), and triangles represent PCV10-vaccinated otitis-prone cases (closed triangles) and non-otitis-prone controls (open triangles). Statistical analysis was conducted on adjusted data, correcting for age and day-care attendance, where **: *p* < 0.01, *: *p* < 0.05 and ns: not significant. NTHi, nontypeable *Haemophilus influenzae*; PCV, pneumococcal conjugate vaccine.

Of the children who were colonised with NTHi, there was no difference in NTHi carriage densities between PCV7- and PCV10-vaccinated otitis-prone (*p* = 0.563) and non-otitis-prone children (*p* = 0.513) (Figure 1).

### 3.4. PCV10-Vaccinated Otitis-Prone Children Had Higher Pneumococcal Nasopharyngeal Densities than PCV7-Vaccinated Otitis-Prone Children

Of the cases who were colonised with *S. pneumoniae*, higher densities of *S. pneumoniae* were detected in the nasopharynx of PCV10-vaccinated otitis-prone children than in PCV7-vaccinated otitis-prone children (*p* = 0.003) (Figure 1). This was not observed in the non-otitis-prone children, where there was a lower (but non-significant) density of *S. pneumoniae* in the nasopharynx of PCV10-vaccinated children compared with the PCV7-vaccinated controls (*p* = 0.074).

### 3.5. Otitis-Prone Children Had Higher Densities of NTHi and S. Pneumoniae in Their Nasopharynx Compared with Non-Otitis-Prone Children

Otopathogen presence and density were compared between the otitis-prone cases and non-otitis-prone controls within each vaccine group. In the PCV7-vaccinated group, otitis-prone cases were more than twice as likely to be colonised with NTHi (aOR = 2.095) and 62.9% more likely to be colonised with *S. pneumoniae* (aOR = 1.629) than the non-otitis-prone controls, *p* = 0.018 and *p* = 0.117 respectively. In the PCV10-vaccinated group, cases were 33.3% more likely to be colonised with NTHi (aOR = 1.333) and 82.3% more likely to be colonised with *S. pneumoniae* (aOR = 1.823) than the non-otitis-prone controls, *p* = 0.396 and *p* = 0.067 respectively. Of the children that were colonised with NTHi, there was no difference in NTHi density in the nasopharynx between cases and controls in the PCV7-vaccinated (*p* = 0.985) and PCV10-vaccinated (*p* = 0.692) groups (Figure 1). In those colonised with *S. pneumoniae*, there was a trend towards higher *S. pneumoniae* densities in the nasopharynx of otitis-prone cases compared to non-otitis-prone controls in the PCV7-vaccinated group (*p* = 0.068), which was even more apparent in the PCV10-vaccinated group (*p* = 0.017) (Figure 1).

### 3.6. Otopathogen Density in the Middle Ear of Otitis-Prone Children was Similar between Vaccine Groups

**Figure 2:**
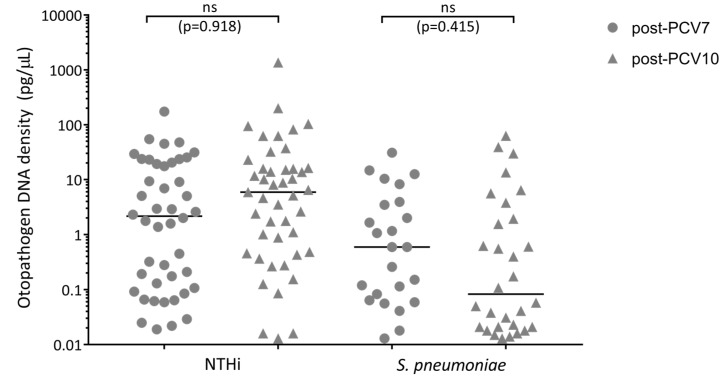
Otopathogen density in the middle ear of PCV7- and PCV10-vaccinated otitis-prone children with pneumococcal or NTHi OM. Data are presented for each child with an otopathogen detected in their middle ear, with each data point representing an individual child and the horizontal bars depicting the median otopathogen DNA concentration in pg/μL in the middle ear effusion. Circles represent PCV7-vaccinated otitis-prone cases and triangles represent PCV10-vaccinated otitis-prone cases. Statistical analysis was conducted on adjusted data, correcting for age and day-care attendance, ns: not significant. NTHi, nontypeable *Haemophilus influenzae*; PCV, pneumococcal conjugate vaccine.

Middle ear effusion was not available for 35 of the 147 PCV7-vaccinated cases and 62 of the 178 PCV10-vaccinated cases. The proportion of children with NTHi detected in their middle ear effusion was 29.9% (44/112) for PCV7-vaccinated cases and 25.8% (46/116) for PCV10-vaccinated cases, *p* = 0.894 (aOR = 0.963). The proportion of children with *S. pneumoniae* detected in their middle ear effusion was 17.0% (25/112) for PCV7-vaccinated cases and 17.4% (31/116) for PCV10-vaccinated cases, *p* = 0.278 (aOR = 1.418). Of the children with NTHi or *S. pneumoniae* detected in their middle ear, there was no difference between otopathogen densities in the middle ear effusion from PCV7- versus PCV10-vaccinated otitis-prone children (NTHi *p* = 0.918; *S. pneumoniae p* = 0.415) (Figure 2).

### 3.7. Correlation between Otopathogen Density in the Nasopharynx and Middle Ear

**Figure 3:**
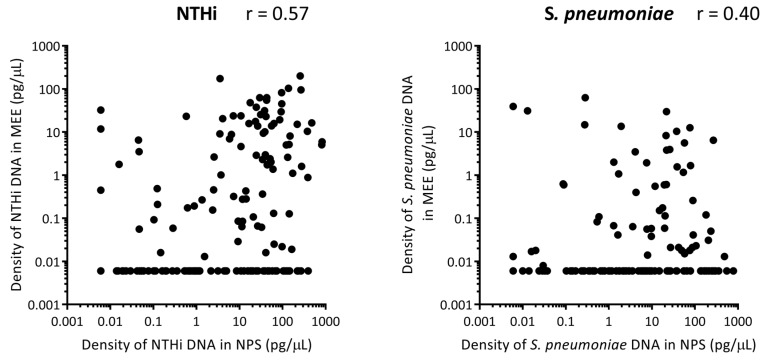
Correlation between otopathogen densities in the nasopharynx with otopathogen density in the middle ear of otitis-prone children. Each dot represents the density of nontypeable *Haemophilus influenzae* (NTHi) (**A**) and *S. pneumoniae* (**B**) in pg/μL of DNA in the nasopharynx (NPS) and middle ear effusion (MEE) of otitis-prone children. Correlation was assessed by Spearman rho, where 0.5 > r >0.3 is considered a weak positive correlation and 0.7 > r >0.5 is considered a moderate positive correlation.

For all of the otitis-prone cases, there was a moderate positive correlation between density of NTHi in the nasopharynx and density of NTHi in the middle ear (*r* = 0.57) (Figure 3). For *S. pneumoniae* there was a weak positive correlation between density in the nasopharynx and middle ear (*r* = 0.40) (Figure 3).

Only 3/325 otitis-prone children (0.9%) had NTHi detected in their middle ear and not their nasopharynx, while 73/325 otitis-prone children (22.5%) had NTHi detected in their nasopharynx but not their middle ear. Likewise, 2/325 (0.6%) otitis-prone children had *S. pneumoniae* detected in their middle ear but not their nasopharynx, and 80/325 (24.6%) otitis-prone children had *S. pneumoniae* detected in their nasopharynx but not their middle ear. ROC curves to assess the diagnostic value of measuring otopathogen density in nasopharyngeal swabs to distinguish otitis-prone from non-otitis-prone children determined the AU-ROC to be 0.63 for NTHi and 0.62 for *S. pneumoniae*, values that are considered to be indicative of a test with sufficient diagnostic ability.

### 3.8. Carriage Density of Pneumococcal Vaccine and Non-Vaccine Serotypes was Similar between All PCV7- and All PCV10-Vaccinated Children

**Figure 4:**
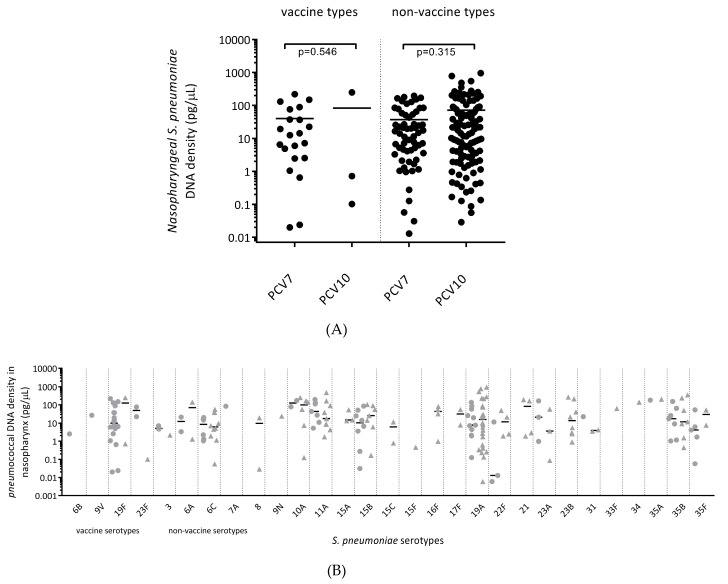
(**A**) Pneumococcal vaccine and non-vaccine serotype carriage density in the nasopharynx of PCV7- and PCV10-vaccinated children. Data are presented for each individual child, with the horizontal bars depicting the median density of vaccine-types and non-vaccine types in pg/μL of pneumococcal DNA. (**B**) Serotype-specific carriage density in the nasopharynx of PCV7- and PCV10-vaccinated children. Data are presented for each individual child, with the horizontal bars depicting the median density of specific pneumococcal serotypes in pg/μL of pneumococcal (*lyt*A) DNA. Circles represent PCV7-vaccinated children and triangles represent PCV10-vaccinated children.

Of all the children who were colonised with *S. pneumoniae*, there was no difference between the nasopharyngeal densities of vaccine types (VT) (*p* = 0.546) and non-vaccine types (NVT) (*p* = 0.315) between PCV7- and PCV10-vaccinated children (Figure 4A). For specific pneumococcal serotypes, nasopharyngeal colonisation with VTs 6B, 9V, 19F and 23F was observed in both vaccine groups (cases and controls combined), but this was more common in the PCV7-vaccinated group (Figure 4B). VT serotype 19F was a common coloniser in the PCV7-vaccinated cohort (18/82 children versus 2/120 in the PCV10-vaccinated cohort), while colonisation with vaccine-related serotype 19A was common in both vaccine groups and often at a high density.

## 4. Discussion

NTHi and *S. pneumoniae* are common bacterial pathogens isolated in upper and lower respiratory tract infections, including OM. With the inclusion of Protein D from NTHi, PCV10 has the potential to offer additional protection against NTHi colonisation and/or disease in comparison with PCV7 (as well as the additive protection against three extra pneumococcal serotypes). In this study, we observed that PCV10-vaccinated otitis-prone and non-otitis-prone children had similar, or even higher, densities of NTHi and *S. pneumoniae* in their nasopharynx when compared with PCV7-vaccinated children. The density of pneumococcal VT and NVT in the nasopharynx was not different between vaccine groups, however there was a significant decrease in the proportion of children colonised with vaccine serotypes in the PCV10 era. It is important to highlight that while VT 6B and 19F have been indicated to elicit some cross-protection against NVT 6A and 19A respectively [32,33], colonisation with both 6A and 19A was observed in both vaccine groups. Although vaccination with PCVs may not reduce the overall carriage density of *S. pneumoniae*, elimination of serotypes with enhanced capacity to cause disease will lead to reduced incidence of acute and complex OM [34]. Indeed, rates of hospitalisation for OM have declined by 25% in New Zealand since introduction of PCV7 in 2006 [35], indicating that while PCVs may be having a limited impact on otopathogen colonisation density, they are having an impact on hospitalisation for OM.

In our study, we did not observe a reduction of NTHi in the middle ear of PCV10-vaccinated otitis-prone children compared with PCV7-vaccinated children. This is in contrast to a recent animal study demonstrating that mice vaccinated with Protein D had reduced NTHi density in the middle ear compared with unvaccinated mice in a model of NTHi AOM [25]. It is possible that we may have missed any short-term impact of PCV10 vaccination in children in our cross-sectional study where middle ear effusion was only collected at the time of surgery, which was months or even years after PCV10 vaccination. PCV-induced antibody responses have not been measured in this study. We have previously shown that Australian otitis-prone children have similar antibody responses to PCV vaccination when compared to non-otitis prone children [36,37,38]. We have also observed that Aboriginal otitis-prone children have similar natural antibody titres to pneumococcal protein antigens when compared with their non-Aboriginal counterparts [39]. Aboriginal otitis-prone children did, however, have lower natural antibody titres to specific NTHi protein antigens [40], indicating that Aboriginal children may be tolerised to NTHi from a young age and cease responding to some NTHi antigens. It is possible that the different ethnic groups within the PCV10 group have different antibody responses to Protein D, but this had no impact on density of NTHi (or pneumococcal) colonisation.

Reports on the impact of Protein D-conjugated pneumococcal vaccines on NTHi colonisation are mixed, and methods used to assess prevalence are variable: studies in Dutch and Finnish children have reported no impact of PCV10 on NTHi carriage density (by qPCR) [21,22], while an increase in NTHi carriage prevalence from 26% to 44% was observed following PCV10 introduction in Brazil [41]. Importantly, apart from the initial trial with a prototype of PCV10 (11 serotypes all conjugated to Protein D) in the Czech Republic [42], there are no reports that have shown a decrease in NTHi carriage prevalence following PCV10-vaccination. In terms of OM, a recent study in Australian Aboriginal children demonstrated that PCV10 vaccination significantly reduced the prevalence of NTHi in ear discharge [20]. Measuring the density of otopathogens in the nasopharynx and middle ear with molecular techniques gives more insight into subtle changes that may be influenced by vaccination.

We and others have previously shown that otitis-prone children have higher densities of otopathogens in their nasopharynx than non-otitis-prone children [24]. Obtaining samples from the site of infection, such as the middle ear, is an invasive and challenging procedure, so the use of nasopharyngeal swabs as a marker of disease aetiology and for monitoring the impact of interventions is appealing. Our data shows a moderate correlation between densities of NTHi in the nasopharynx and middle ear of otitis-prone children, suggesting that detection of NTHi in nasopharyngeal swabs is indicative of NTHi-associated OM, but cannot be used as a definitive test. Only a weak correlation was observed for *S. pneumoniae* densities in the nasopharynx and middle ear of otitis-prone children, suggesting that nasopharyngeal swabs are not a good indication of pneumococcal OM diagnosis, as has been concluded by studies assessing the utility of nasopharyngeal swabs as a proxy for pneumococcal pneumonia aetiology across a wide range of settings [43,44].

Over-representation of day-care attendance in otitis-prone children is well documented [45,46,47], and shows that our cohort is representative of cohorts in other countries. Day-care attendance in the PCV10-vaccinated group was higher than the PCV7-vaccinated group; this may be linked to the slightly older age range in the PCV10 group, but is more likely to have been influenced by the change in government policy in 2012 to provide 20 hours of free childcare for all New Zealand children [48]. To ensure that only otopathogen density was compared between vaccine groups, the confounding factors of age and day-care were adjusted for in all analyses. The PCV10-vaccinated group was recruited 3 years after the PCV7-vaccinated group, and 6 years after PCV7 introduction into New Zealand, which may have impacted the circulating pneumococcal serotypes in the population. Longitudinal studies with frequent sampling may reveal short-lived temporal changes in otopathogen density in the nasopharynx or middle ear following PCV10-vaccination, but ideally, any impact on carriage density should be sustained. In addition, prolonged (3 years) cryopreservation of the specimens from the PCV7 era may have led to a lower otopathogen density compared with the specimens from the PCV10 era. However, studies have shown no reduction in viability counts over a >10 year period for both *S. pneumoniae* and NTHi cultured from nasopharyngeal swabs collected in STGGB media, which is the same media used in this study [49,50].

## 5. Conclusions

In summary, 3 years following the introduction of PCV10 into New Zealand, there was no reduction in NTHi or *S. pneumoniae* density in the nasopharynx of otitis-prone or non-otitis-prone children. There was also no difference in otopathogen density in the middle ear of PCV10-vaccinated otitis-prone children compared with PCV7-vaccinated otitis-prone children. Development of improved strategies to reduce otopathogen density in the nasopharynx, and their dissemination into the middle ear, are warranted in attempt to reduce the burden of OM.

## Figures and Tables

**Table 1:** qPCR primers, probes and conditions.

Assay	Detected Species	Primer/Probe ^a^	Sequence (5’ to 3’)	Concentration in Reaction Mix	Cycling Conditions	Ref.
*fuc*P	*H. influenzae*	*fuc*P fwd	GCCGCTTCTGAGGCTGG	1000 nM	50 °C for 2 min and 95 °C for 10 min, followed by 40 cycles of 95 °C for 15 sec and 60 °C for 60 sec.	[26]
		*fuc*P rev	AACGACATTACCAATCCGATGG	1000 nM		
		*fuc*P probe	6FAM-TCCATTACTGTTTGAAATAC-MGBNFQ	1000 nM		
*hpd#3*	*H. influenzae*	*hpd*3 fwd	GGTTAAATATGCCGATGGTGTTG	1000 nM	50 °C for 2 min and 95 °C for 10 min, followed by 40 cycles of 95 °C for 15 sec and 60 °C for 60 sec.	[27]
		*hpd*3 rev	TGCATCTTTACGCACGGTGTA	1000 nM		
		*hpd*3 probe ^b^	HEX-TTGTGTACACTCCGT/ZEN/TGGTAAAAGAACTTGCAC-3C6	1000 nM		
*lyt*A	*S. pneumoniae*	*lyt*A fwd	ACGCAATCTAGCAGATGAAGCA	200 nM	95 °C for 10 min, followed by 40 cycles of 95 °C for 15 sec and 60 °C for 60 sec.	[28]
		*lyt*A rev	TCGTGCGTTTTAATTCCAGCT	200 nM		
		*lyt*A probe	6FAM-TGCCGAAAACGCTTGATACAG-GGAG-BHQ1	200 nM		

(a) Fwd, forward; Rev, reverse. (b) Probe was modified from [27] with a ZEN internal quencher instead of the internal black whole quencher (BHQ).

**Table 2:** Demographics of study cohort and comparison between cases and controls within PCV7- and PCV10-vaccinated groups.

	PCV7 Vaccine Group			PCV10 Vaccine Group		
Sample Demographics	Cases	Controls	*p*	Cases	Controls	*p*
Total number	147	70		178	62	
Median age in months (interquartile range)	21.34 (16.54–26.63)	18.82 (12.86–24.8)	0.031	24.17 (19.05–28.81)	21.11 (14.7–29.08)	0.043
Male gender (%)	92 (63%)	51 (73%)	0.136	122 (69%)	34 (55%)	0.051
Day care attendance (%)	93 (63%)	28 (40%)	0.002	144 (81%)	28 (45%)	0.0001
Antibiotics in last month (%)	74 (50%)	29 (41%)	0.201	78 (44%)	18 (29%)	0.171
*Ethnicity (%)*						
European	104 (71%)	45 (64%)	0.337	103 (58%)	30 (48%)	0.196
Māori	21 (14%)	5 (7%)	0.130	45 (25%)	14 (23%)	0.671
Pacific Island	19 (13%)	13 (19%)	0.273	27 (15%)	9 (15%)	0.901
Other/unknown	3 (2%)	7 (10%)	0.014	3 (2%)	9 (15%)	0.0001

**Table 3:** Comparison of demographics for otitis-prone cases and non-otitis-prone controls between vaccine groups.

	Otitis-Prone Children (Cases)			Non-Otitis-Prone (Controls)		
Sample Demographics	PCV7	PCV10	*p*	PCV7	PCV10	*p*
Total number	147	178		70	62	
Median age in months (interquartile range)	21.34 (16.54–26.63)	24.17 (19.05–28.81)	0.007	18.82 (12.86–24.8)	21.11 (14.7–29.08)	0.183
Male gender (%)	92 (63%)	122 (69%)	0.260	51 (73%)	34 (55%)	0.031
Day care attendance (%)	93 (63%)	144 (81%)	0.001	28 (40%)	28 (45%)	0.270
Antibiotics in last month (%)	74 (50%)	78 (44%)	0.176	29 (41%)	18 (29%)	0.325
*Ethnicity (%)*						
European	104 (71%)	103 (58%)	0.016	45(64%)	30 (48%)	0.066
Māori	21 (14%)	45 (25%)	0.014	5 (7%)	14 (23%)	0.012
Pacific Island	19 (13%)	27 (15%)	0.564	13 (19%)	9 (15%)	0.533
Other/unknown	3 (2%)	3 (2%)	0.813	7 (10%)	9 (15%)	0.428
